# Leveraging AI tools for streamlined library event planning: a case study from Lane Medical Library

**DOI:** 10.5195/jmla.2025.2087

**Published:** 2025-01-14

**Authors:** Boglarka Huddleston, Colleen Cuddy

**Affiliations:** 1 b.huddleston@stanford.edu, Manager, Research & Instruction, Lane Medical Library, Stanford University School of Medicine, Stanford, MA; 2 ccuddy@stanford.edu, Director, Research and Academic Collaboration, Lane Medical Library, Stanford University School of Medicine, Stanford, MA

**Keywords:** Artificial Intelligence, event planning, medical library

## Abstract

Health sciences and hospital libraries often face challenges in planning and organizing events due to limited resources and staff. At Stanford School of Medicine's Lane Library, librarians turned to artificial intelligence (AI) tools to address this issue and successfully manage various events, from small workshops to larger, more complex conferences. This article presents a case study on how to effectively integrate generative AI tools into the event planning process, improving efficiency and freeing staff to focus on higher-level tasks.

## BACKGROUND AND CONTEXT

In the past year, Lane Medical Library organized a series of events using AI tools to assist with planning. Individual or small teams of librarians typically are responsible for event content and they work with the library marketing and communications team to promote events. Librarians are responsible for planning and executing events from start to finish. Once speaker(s), date, location, and event program are confirmed, the marketing team creates promotional materials that are distributed via different channels (newsletters, listservs, social media, etc.). The marketing and communications team is comprised of three staff members, led by and including the library's Web Services Librarian. Staff (librarians and/or library staff) rotate through the team on an annual basis.

Recently, two notable events took place where event organizers heavily relied on the assistance of AI tools: 1) a live, online conversation with the co-founder of MedRx with 35 participants in attendance; and 2) a half-day online conference with over 250 participants titled “Women in Data Science: Artificial Intelligence and Health Equity”. While these events differed in complexity, both benefited from AI support, which streamlined various aspects of the planning and execution process.

## EVENT PLANNING WITH AI

Regardless of event size and complexity, the event planning process can be divided into three stages: pre-event, during the event, and post-event. Each stage offers unique opportunities to leverage AI tools to improve efficiency and reduce manual workload.

**Figure 1:**
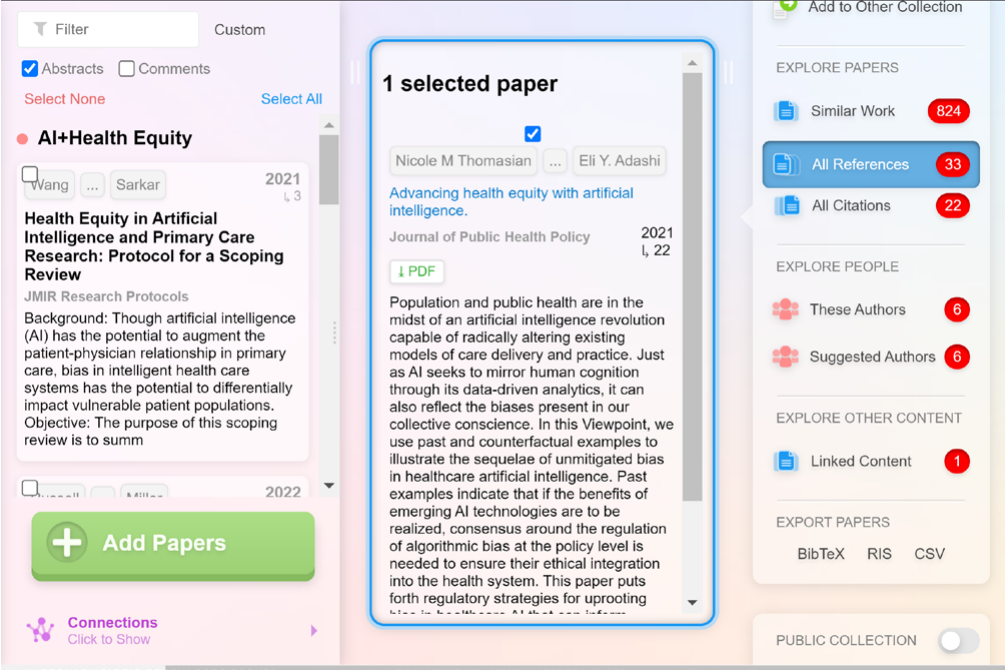
Selecting sample paper in ResearchRabbit

**Figure 2:**
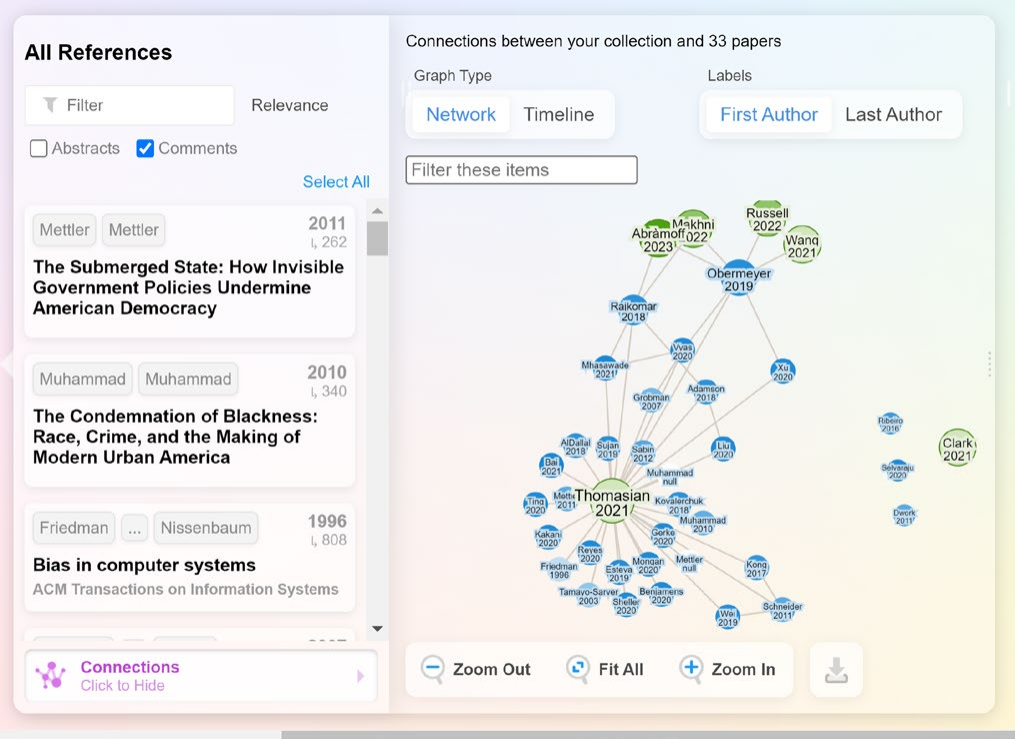
Visualizing connections to relevant papers in ResearchRabbit

*Pre-Event:* During the pre-event phase, event organizers used AI tools to help manage content, structure planning, and marketing. For instance, Lane ResearchRabbit, an AI tool that allows users to search for papers and authors, monitor new literature, and visualize research landscapes, to identify potential speakers by generating interactive visualizations of relevant networks of papers and authors significantly reduced the time required to find and vet speakers for the events (see Figure 1 and Figure 2).

Additionally, event organizers employed ChatGPT 4.0 to create show-flow agendas, event pacing, speaker introductions, and panel questions, saving several manual work hours.

**Figure 3:**
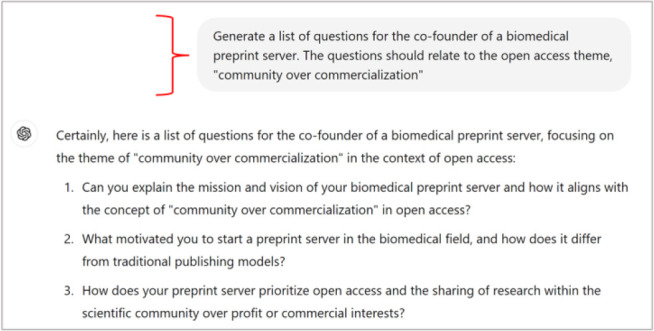
ChatGPT 4.0 Prompt for panel questions

*During the Event*: During live events, event organizers relied on ChatGPT 4.0 to generate on-the-fly panel discussion questions, allowing staff to focus on facilitating the event and engaging with participants.

*Post-Event:* After each event, ChatGPT 4.0 assisted in crafting follow-up communications, including thank-you emails to speakers and participants. The event organizers also used AI to generate post-event surveys, helping gather feedback to refine future events.

## CONCLUSION

These experiences have demonstrated the benefits of generative AI tools for event planning, particularly in automating repetitive tasks and reducing time spent on event logistics, allowing organizers to focus on content creation and community engagement. However, event organizers also encountered a few challenges. AI-generated content sometimes included inaccuracies or overly complex language that required human intervention. In these cases, they had to manually revise speaker bios that contained incorrect information and streamline AI-generated email templates that contained superfluous content. This required fact-checking, for example, reviewing content against online profiles or LinkedIn to verify speaker information and some judicious editing for communications. Staff learned to create well-structured, clear AI prompts, through trial and error and sharing prompt libraries, which led to more accurate, relevant outputs, minimizing errors and misinterpretations. In the end, this improved efficiency, saved time, and enabled faster completion of tasks, thus enhancing overall productivity in the workflow.

AI tools have greatly enhanced event planning capabilities at Lane Medical Library, particularly in streamlining complex tasks and automating routine processes. While these tools require careful oversight and occasional adjustments, they have enabled library staff to deliver high-quality events more quickly and efficiently. For other libraries facing similar challenges, the Lane Library recommends exploring AI tools to augment traditional planning processes and maximize available resources.

